# The Role of 5-Phosphodiesterase Inhibitors (PDE-5I) in Current Benign Prostatic Hyperplasia Treatment: A Narrative Review

**DOI:** 10.3390/medicina60111736

**Published:** 2024-10-23

**Authors:** Konstantinos Stamatiou, Gianpaolo Perletti, Vittorio Magri, Alberto Trinchieri

**Affiliations:** 1 Urology Department, Tzaneio General Hospital, 18536 Piraeus, Greece; 2 Department of Biotechnology and Life Sciences, Section of Medical and Surgical Sciences, University of Insubria, 21100 Varese, Italy; gianpaolo.perletti@uninsubria.it; 3 Urological Clinic, ASST North, 20026 Milan, Italy; vittorio.magri@virgilio.it; 4 Department of Urology, IRCCS Ca’ Granda Ospedale Maggiore Policlinico, University of Milan, 20122 Milan, Italy; alberto.trinchieri@gmail.com

**Keywords:** benign prostate hypertrophy, lower urinary tract symptoms, bladder outlet obstruction, PDE-5 inhibitor, alpha1-adrenergic antagonist, 5-alpha-reductase inhibitors

## Abstract

*Introduction*: 5-phosphodiesterase inhibitors (PDE-5I) have been investigated as a treatment for urinary dysfunction for almost a decade. The general perception is that they play a significant role in managing lower urinary tract symptoms (LUTS), particularly those associated with benign prostatic hyperplasia (BPH). However, the specific biochemical processes by which PDE-5I repairs urinary function are still poorly understood and there is little instrumental evidence of significant improvement in urinary symptoms. Therefore, we explore the role of 5-phosphodiesterase inhibitors (PDE-5I) as complementary to the conventional treatment of symptomatic BPH; we provide the suggested biological procedures involved in the association between PDE-5 inhibitor use and improvement in LUTS; and we propose new approaches to this topic. *Material and Methods:* A systematic search for clinical trials, experimental studies, and systematic reviews was performed in electronic libraries (PubMed, EMBASE, Scopus) using the terms “benign prostate hypertrophy”, “benign prostate hyperplasia”, “lower urinary tract symptoms”, “storage symptoms”, “voiding symptoms”, “bladder outlet obstruction” and the keywords “mechanism of action”, “synergy”, “PDE-5 inhibitor”, “alpha1-adrenergic antagonist”, “5-alpha-reductase inhibitors” in various combinations. There was no restriction on publication date. *Results*: To date, only a few randomized studies have been published in which the effect of the combination of a conventional drug for the treatment of symptomatic BPH and a PDE-5I was investigated. Almost all showed significant improvement in IPSS and QoL. Some studies showed significant improvements in maximum urine flow (Qmax) and postvoiding residual volume (PVR) with combination therapy compared with a single agent alone. *Conclusions*: PDE-5I seems effective in relieving symptoms of some BPH patients when administered as complementary to agents currently used to treat BPH. However, the mechanism of action of PDE-5 inhibitors in LUTS remains poorly understood and it is difficult to determine the specific subset of BPH patients who will benefit from the combination of PDE-5 inhibitors with the current treatment. Well-designed, sufficiently informative comparative studies focusing on specific target group profiles (age, urogenital parameters) are needed to define new therapeutic options.

## 1. Introduction

Benign prostate hyperplasia (BPH) is present in almost half of men over 50 years of age. Less than half of all men with BPH suffer from a variety of symptoms of the lower urinary tract (LUTS), consisting of increased voiding frequency, hesitancy, nocturia (storage/irritative symptoms), weak stream, sense of incomplete emptying, intermittent stream (voiding/obstructive symptoms), which may or may not accompany urinary incontinence and some degree of erectile dysfunction (ED) [1]. The pathophysiology and the physical development of BPH-related LUTS are not fully understood. According to current perspectives, they can be attributed to both static elements, such as bladder outlet obstruction, secondary to BPH-induced compression of the urethra, and dynamic elements, such as destabilization of bladder smooth muscle tone owing to denervation hypersensitivity of the bladder’s neuromuscular structures [2]. Some authors suggest a shared pathophysiological mechanism associating BPH-induced ED and LUTS. This mechanism includes dysfunction of the microvasculature of the bladder, the prostate, the urethra, and the corpus cavernosum as well, along with local neural malfunction that triggers a cascade of hypoxemia, vasoconstriction, alteration of the contractile function of the local musculature, and finally degradation of the autonomic neurons and ganglia function [3].

Today’s pharmaceutical treatments for BPH-related LUTS include several options aimed at relieving symptoms and improving quality of life. The main categories of medications in use are alpha1-adrenoceptor antagonists (alpha-blockers, AB), 5-alpha-reductase inhibitors (5ARI), anticholinergic (antimuscarinic) agents, beta-3 agonists, 5-phosphodiesterase inhibitors (PDE5-I), and combinations of the above.

The concept behind the use of AB is that prostate enlargement secondary to BPH results in a considerable increase in the overall number of alpha1-adrenoceptors, which causes increased stromal smooth muscle tone. These drugs relax the smooth muscle tone by inhibiting alpha1-adrenoceptors of the prostate and bladder neck, thus relieving urethral obstruction and the associated voiding symptoms [4].

Adverse effects associated with AB treatment include hypotension, dizziness, headache weakness, tachycardia, and tremulousness. Adverse effects tend to occur more often in the elderly and increase the risk of falls who receive nonselective alpha-blockers [4].

5ARI improves symptoms by reducing the size of the prostate by blocking the action of the 5-alpha-reductase enzyme, which catalyzes the conversion of the main male sex hormone testosterone into the more potent androgen dihydrotestosterone. It is believed that the increased gland volume causes a mechanical compression of the prostatic urethra. Often, a hypertrophic median lobe protrudes in the bladder lumen, creating an additional obstacle in the bladder outflow. The 5ARI-induced decrease in the size of the prostate can shrink the median lobe and decompensate the prostatic urethra. Simultaneously, a significant reduction in the overall number of alpha1-adrenoceptors also occurs [5].

Reported side effects of 5ARIs include sexual dysfunction, infertility, mood disorders, gynecomastia, high-grade prostate cancer, breast cancer, and cardiovascular morbidity.

The combination of a 5ARI and an AB significantly reduces obstructive symptoms and the clinical progression of BPH compared with single-agent therapy with drugs of either class.

For this reason, this combination is currently used in patients with symptoms refractory to ABs for more effective symptom management.

Anticholinergic (antimuscarinic) agents are primarily used to treat overactive bladder syndrome. These drugs block the effect of the neurotransmitter acetylcholine on the muscarinic receptors in the central and peripheral nervous systems. Their action at the level of the bladder results in the relaxation of the fibers responsible for the involuntary movement of the detrusor [6]. Experimental evidence suggests that muscarinic receptors promote the growth of prostate tissue and receptor inhibition induced by anticholinergic agents may potentially impede prostate enlargement [7].

Nowadays, combination therapy with AB and antimuscarinic agents is indicated when the bladder outlet obstruction related to BPH coexists with overactive bladder symptoms. It should be noted that treatment with antimuscarinics is associated with bothersome side effects including constipation, bradycardia, tachycardia, palpitations, arrhythmias, reduced bronchial secretions, urinary retention, photophobia, dry mouth, flushing, and dryness of the skin.

Beta-3 agonist drugs are a relatively new class of medications primarily used to treat obesity. Today, they are indicated to treat overactive bladder syndrome (OAB). These drugs activate beta-3 adrenergic receptors in the bladder to couple with Gs protein. This leads to increased activity of the enzyme adenylyl cyclase, which subsequently increases the formation of cyclic adenosine monophosphate (cAMP). Accumulation of cAMP leads to a decrease in the level of stimulation of the smooth muscles, a reduction in the frequency of the contractions, and relaxation of the detrusor muscle fibers. It is also possible that beta-3 agonist drugs affect the detrusor muscle indirectly by promoting endothelial nitric oxide synthase (eNOS) activity and NO bioavailability. As a consequence, the bladder’s functional capacity increases, allowing it to deposit a greater amount of urine, reducing the urgency and frequency of urination [8].

Treatment with Beta-3 agonist is associated with bothersome side effects including elevated blood pressure that occurs mainly in persons with preexisting hypertension, dry mouth, bronchitis nasopharyngitis, sinusitis, rhinitis, headache, back pain, arthralgia, diarrhea, nausea, constipation, dyspepsia, gastritis and gastroenteritis, blurred vision, glaucoma, dizziness, fatigue, tachycardia, atrial fibrillation, palpitations, and hot flush.

Both anticholinergic (antimuscarinic) agents and beta-3 agonists can be taken in combination with other drugs to enhance their effectiveness. As these drugs help relax the bladder muscle, they potentially provide additional symptom relief in patients suffering from symptomatic BPH.

Phosphodiesterase type 5 inhibitors (PDE5-Is) are a class of drugs primarily used to treat erectile dysfunction (ED) and pulmonary hypertension. They work by blocking the enzyme PDE-5, which results in the relaxation of smooth muscle cells and increased blood flow to specific areas of the body. For almost a decade, PDE5-Is were investigated for treating urinary dysfunction. Currently, the general perception is that they play a significant role in managing LUTS, particularly those associated with BPH.

The side effects associated with PDE5-Is are generally mild. More common are dizziness, flushing, dyspepsia, nasal congestion, and rhinitis. Patients taking tadalafil have reported back pain and muscle aches.

Evidence suggests that combination therapy with AB and PDE5-I enhances the activity of both agents, providing extra relaxation of the smooth muscle tone and repairing BPH-related ED [9]. However, the specific processes by which PDE5-I repairs urinary function are still poorly understood, and it remains unclear to what extent complementary PDE5-I treatment adds benefit to the current BPH treatment. According to the existing literature, there is little instrumental evidence of significant improvement in urinary symptoms. Therefore, it is important to determine the specific subset of BPH patients who will benefit from the combination of PDE-5 inhibitors with agents currently used to treat BPH.

Therefore, we explore the role of PDE5-Is as complementary to the conventional treatment of symptomatic BPH; we provide the suggested biological procedures involved in the association between PDE5-I use and improvement in LUTS; and we propose new approaches to this topic.

## 2. Materials and Methods

A systematic search for randomized clinical trials was performed in electronic libraries (PubMed, EMBASE, Scopus) using the terms “benign prostate hypertrophy”, “benign prostate hyperplasia”, “lower urinary tract symptoms”, “storage symptoms”, “voiding symptoms”, “bladder outlet obstruction” “overactive bladder syndrome” and the keywords “mechanism of action”, “biochemical processes”, “synergy”, “5-phosphodiesterase inhibitors”, “alpha1-adrenergic antagonist”, “antimuscarinic agents”, “beta-3 agonists”, in various combinations. Literature information in the selected publications was further checked for relevant publications not included in the initial search. We evaluated experimental studies, review articles, and meta-analyses to provide accurate conclusions. There was no restriction on the publication date. In order to better understand the complementary role of 5-phosphodiesterase inhibitors (PDE-5Is) in the current benign prostatic hyperplasia treatment, we designed a pooled summary of the most well-designed randomized controlled trials, comparing the effect of therapy of BPH patients with a combination of alpha1-adrenoceptor antagonists (AB) and 5-phosphodiesterase inhibitors (PDE-5Is) vs. AB plus placebo.

## 3. Results

**Figure 1:**
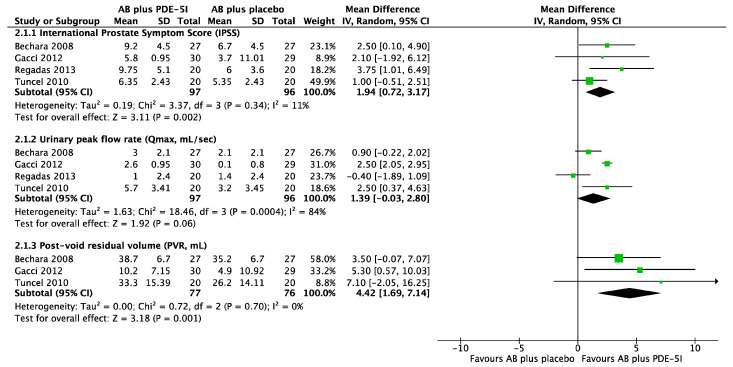
Pooled summary of 4 randomized controlled trials included in this review, comparing the effect of therapy of BPH patients with a combination of alpha1-adrenoceptor antagonists (AB) and 5-phosphodiesterase inhibitors (PDE-5I) vs. AB plus placebo. Forest plots indicate the mean difference of pre-post treatment improvements of (i) IPSS total scores, (ii) peak urinary flow (Qmax) and (iii) post-void residual volume (PVR). Data to the right of the vertical no-effect line of forest plots represent a favorable effect of combination therapy versus single-agent plus placebo. Diamonds represent overall effect sizes extending to the limits of the 95% confidence intervals of mean differences. The significance of the pooled effect sizes (Z statistics) and heterogeneity data (I^2^, Chi-square, *p* value) are shown. Inverse variance statistics, random effects model. RevMan 5.3. When not disclosed in study reports, standard deviations were calculated using the imputeSD package on the R platform. Bechara 2008 is [9], Gacci 2012 is [10], Regadas 2013 is [11], Tuncel 2010 is [12].

There is a relative lack of large, randomized trials investigating the role of PDE5-Is as complementary to the conventional treatment of symptomatic BPH (Figure 1). In existing studies, the following comparisons have been tested: PDE5-I and AB versus AB alone [9,10,11,12,13], PDE5-I and AB versus PDE5-I alone [11,13,14], PDE5-I versus AB versus PDE5-I plus AB [15,16], PDE5-I versus AB as combination therapy with 5-ARI [17], and PDE5-I and 5-ARI versus 5-ARI alone [18]. Co-administration of a standard dose of 5-ARI (dutasteride 0.5 mg/day) and variable doses of PDE5-I (tadalafil 2.5 mg/day, 5 mg/day and 20 mg/day) and an anticholinergic agent (solifenacin 2.5 mg/day, 5 mg/day and 10 mg/day) was also evaluated [19]. Finally, two recent studies examined the efficacy of PDE5-I add-on therapy in patients receiving 5-ARI and that of PDE5-I monotherapy versus a combination of PDE5-I and a beta-3 adrenoceptor agonist [20,21].

The combination of PDE-5I and AB has been proven to be more effective in terms of IPSS total score reduction and improved voiding signs than PDE-5I and AB administered as a single therapy (Table 1). The superiority of the PDE-5I/AB combination over AB alone persisted in the long-term follow-up (13 to 26 weeks). Comparisons between PDE5-I and 5-ARI versus 5-ARI alone also revealed some improvement in the IPSS total score in the combination arm (Figure 1 and Table 1). However, only one study showed a significant increase in maximum urine flow (Qmax) and a decrease in postvoiding residual volume (PVR) with combination therapy compared with a single agent alone [19]. Whether PDE5-I/AB combination superiority over PDE-5I alone lasts over time is not known, since study results were limited to treatment duration (between 4 and 12 weeks). Similarly, the long-term superiority of the PDE5-I/5-ARI combination versus 5-ARI alone remains unspecified due to the limited length of the follow-up (up to 12 weeks). In confirmation of the above, one study showed that the AB/5-ARI combination provides significantly better Qmax after the fourth week of treatment [17]. Of note, 5-ARI with PDE5-I add-on therapy provided no improvement regarding maximum urine flow and residual urine volume (Table 1) [20].

Regarding storage symptom relief, the effect of PDE-5I monotherapy appears to be lower than that of either PDE5-I/beta-3 adrenoceptor and PDE5-I/anticholinergic agent combination [16,21]. It should be mentioned that the total assessment of symptoms of hyperactivity significantly decreased in patients who received the higher dose of both PDE5-I/anticholinergic drugs after the fourth week and remained below the baseline until the end of the study [19].

Adding a PDE5-I to conventional BPH treatment can have several mild adverse effects which rarely are problematic for some patients. Some of the most frequently reported side effects include hypotension, headaches, flushing (redness or warmth in the face, neck, or chest), indigestion or stomach discomfort, nasal congestion, visual disturbances (changes in vision, such as blurred vision or a blue tint), back pain and muscle aches. These can occur, particularly with higher doses. Side effects were more frequent in patients treated with the PDE5-I/AB combination compared to patients treated with AB alone. No difference in rates of adverse effects between patients treated with the PDE5-I/5-ARI combination and patients treated with 5-ARI alone was noted.

## 4. Discussion

At present, the combination of a conventional drug for the treatment of symptomatic BPH and a PDE5 inhibitor is not routinely offered for the treatment of BPH-associated LUTS [22]. Evidence of a clear improvement in urination with the addition of PDE5-I to 5-ARI or AB is somehow controversial, given that improvements assessed in patient questionnaires may be subjective and may be affected by recall biases. Moreover, some evidence from published studies is limited to short-term treatment and short follow-up.

On the other hand, there is a body of evidence from several trials that have shown a consistent and strong relationship between LUTS and ED. Actually, BPH can contribute to erectile dysfunction due to the pressure the enlarged prostate puts on the urethra and surrounding tissues. Moreover, many men with BPH experience a significant decrease in sexual satisfaction and libido. This can be due to both physical discomfort and psychological stress related to urinary symptoms [23]. In such a case, conventional drugs for the treatment of symptomatic BPH would be able to reduce BPH-associated ED by reducing the bothersome LUTS. To our knowledge, recent studies showed that in cases where AB treatments have failed, administration or co-administration of a PDE5-I can effectively improve the IPSS urinary storage symptom subscore where alpha blocker treatments have failed [14,24]. It therefore seems clear that the addition of PDE5-I to 5-ARI or AB could be beneficial to the subset of patients suffering from treatment refractory BPH-related ED.

The association between BPH-related LUTS and ED could be coincidental since BPH often coexists with conditions such as diabetes, hyperlipidemia, and atherosclerosis. Alternatively, the above association could be explained within the frame of metabolic syndrome [25]. Moreover, it appears that the pathophysiological mechanisms of LUTS and the related prostatic enlargement of BPH may have an impact on both the erection and ejaculation components of the sexual response [26]. There is considerable evidence demonstrating the activity of PDE5-I in the prostate, the bladder, and the urethra. As a matter of fact, experimental studies demonstrated the presence of functional proteins and mediators of the NO–cGMP also in other districts of the lower urinary tract [27,28,29]. The precise biological process by which the NO–cGMP pathway directly or indirectly affects the micturition reflex and the regulatory role of phosphodiesterases remain poorly understood. However, the fundamental biochemical mechanism modulating the NO/cGMP pathway responsible for corpus cavernosum smooth muscle relaxation has been intensively studied. In brief, increased levels of endothelial NO led to an increase in cGMP production, which subsequently activates a series of phosphorylations and a single dephosphorylation. The latter results in the dissociation of myosin from actin fibers and ultimately in smooth muscle relaxation [30]. Experimental studies simulating PDE5-I activity showed that interruption of the above-mentioned phosphorylation cascade at the level of cGMP production results in prolongation of smooth muscle contraction. In normal rats, inactivation of the NO–cGMP pathway in the lower urinary tract in vivo and in vitro results in the development of bladder overactivity [31]. In genetically altered mice with cGKI-deficiency, inactivation of the NO–cGMP pathway causes irregular cystometrograms characterized by frequent voiding, decreased intercontraction intervals, and non-voiding bladder contractions [32]. Interestingly, Gillespie et al. found subepithelial neurogenic nitric oxide synthase (NOS)—containing nerves immersed among bladder interstitial cells [33]. Though the role of them is not fully investigated, these authors suggest that they produce cGMP to facilitate muscle relaxation. Given that NOS-containing nerves are significantly denser in the outer layer of the bladder, it could be postulated that the NO–cGMP pathway is part of a neuromodulator mechanism involved in the micturition process and that inhibitors of cGMP degradation, namely PDE5-Is, may control bladder function through inhibition of neurotransmission in the bladder afferent nerves [34].

In light of this evidence, the subset of patients suffering mainly from irritative LUTS may benefit from the combination of a conventional drug for the treatment of symptomatic BPH and a PDE5 inhibitor.

Studies have shown that abundant NOS-containing nerves are sited among muscular cells of the urethral wall. In such a case, discontinuation of the NO–cGMP pathway may also affect sites other than the bladder [35]. As a matter of fact, targeted disruption of the *nNOS* gene in experimental models of mice resulted in bladder–urethral sphincter dysfunction, decreased bladder capacity urinary bladder hypertrophy voiding abnormalities, and deficient outflow relaxation [36].

Taking together the above considerations, Mouli and McVary suggested that decreased nitrinergic innervation in BPH, along with a significant reduction in NO-mediated smooth muscle relaxation, may play a key role in the generation of LUTS [37]. In such a case, PDE5-I activity on specific sites of the prostate and bladder may counteract the contraction of the prostate smooth muscle but also that of the bladder neck and urethra, either directly or indirectly. Given the impact of PDE5-Is on urinary bladder hypertrophy, another target group may consist of patients with BPH and bladder trabeculation. The role of PDE5 inhibitors in the management of specific urethral dysfunctions has not been studied yet; therefore, it is difficult to determine whether and if the specific subset of BPH patients with urethral dysfunctions will benefit from the combination of PDE5-Is with agents commonly administered to benign prostatic hyperplasia.

In addition to prolonged contraction of the human prostate smooth muscle, inactivation of the NO–cGMP pathway may be attributed to the development of prostate stromal hyperplasia [38]. In fact, Tinel et al. demonstrated that the NO–cGMP biochemical pathway is able to control smooth muscle cell proliferation through the antiproliferative properties of cGMP [39]. Bloch et al. showed a significant difference in nitrinergic innervation and NOS/NO levels in the transition zone between hyperplastic and normal prostates [40]. Notably, Luo et al. demonstrated an association between a decline in NOS level, age and increased prostate volume [41]. Given the antiproliferative effects of PDE-5Is, it could be hypothesized that patients with progressive BPH in terms of prostate enlargement may also benefit from the addition of a PDE5-I to the ordinary treatment.

Conventional treatments for BPH, such as ABs and 5-ARIs, can sometimes lead to sexual side effects. These may include ED, reduced libido, and decreased ejaculate volume [42]. PDE5-Is play a significant role in addressing sexual side effects caused by conventional BPH treatments by enhancing blood flow to the penis and through improvement of erectile function, positively impacting sexual desire and overall sexual satisfaction. Currently, combination therapy appears appropriate for patients with both LUTS and BPH treatment-related sexual dysfunction.

Besides relaxation of bladder smooth muscle, bladder compliance changes, and improvement in bladder wall perfusion, other possible effects of PDE5-Is on LUTS may involve activities at the level of the central nervous system [43]. The latter may explain why improvements in symptom scores were not evident in urodynamic studies (mainly measuring the maximum urine flow and post-void residual volume) [44]. Further evidence of an alternative PDE5-I activity was demonstrated by Brock et al. who showed that the efficacy of PDE5-I is irrelevant to the erectile function status of the patients [45]. As long as the exact mechanism by which PDE5 inhibitors may improve LUTS remains unclear, it is difficult to determine the specific subset of BPH patients who will benefit from the combination of PDE5-I with agents commonly administered to benign prostatic hyperplasia patients.

## 5. Conclusions

PDE-5I seems effective in relieving symptoms of some BPH patients when administered as complementary to agents currently used to treat BPH. Clinical studies suggest that the combination of ABs or 5ARIs with PDE5-I may improve BPH-associated symptoms when compared with either drug alone. However, a clear improvement in urination remains somehow controversial. In contrast, there is robust evidence of improving persistent urinary storage symptoms. Adverse effects associated with the addition of a PDE-5I to AB treatment are generally mild and well tolerated. Additionally, PDE5 inhibitors can improve erectile function, making them a dual-purpose therapy for men experiencing both BPH and ED. Even so, the mechanism of action of PDE5 inhibitors in BPH-related LUTS has not been clarified and remains poorly understood. Therefore, it is difficult to determine the specific subset of BPH patients who will benefit from the combination of PDE5 inhibitors with agents currently used to treat benign prostatic hyperplasia (BPH). Well-designed, adequately powered comparative trials focusing on specific target population profiles (age, urogenital parameters) are needed to define novel therapeutic opportunities.

## Figures and Tables

**Table 1:** Full experimental details and results in the existing literature.

Autor.	#Ν	Tools	Comparison	Results
Bechara et al. [9]	30	IPSS, IPSS-QoL, Qmax, PVR, GAQ, IIEF-EF,	TMS vs. TMS + TDF	No significant differences in Qmax and PVR. IIEF improvement with TMS + TDF.
Gacci et al. [10]	60	IPSS, IIEF-5, OAB-q, Qmax, Qave, RPV	TMS vs. TMS + VRF vs. placebo	Qmax, Qave, irritative-IPSS, and IIEF better with VRF vs. placebo.
Regadas et al. [11]	40	Pdet-Qmax Qmax, IPSS	TMS + TDF vs. TMS + placebo	TMS + TDF reduces Pdet-Qmax without changing the maximum flow rate during micturition.
Tuncel et al. [12]	60	IPSS, Qmax, PRV,	SLF vs. TMS vs. TMS+ SLF	TMS + SLF is not superior to TMS in enhancing voiding symptoms.
Agenosov et al. [13]	60	IPSS, QoL	TMS vs. TMS + TDF vs. TDF	Significant increase in QoL with TMS + TDF.
Ye et al. [14]	126	IPSS, IIEF-5	TMS + TDF vs. TMS vs. placebo	Both TMS + TDF and TMS alone had similar improvement in IPSS storage symptoms and IIEF-5.
Singh et al. [15]	133	IPSS, IPSS- QoL index, Qmax, and PVR, IIEF-5	TMS vs. TMS + TDF vs. TDF	Monotherapy with either TMS or TDF showed similar results in efficacy endpoints with TMS + TDF.
Abdelrazek et al. [16]	308	Qmax, IPSS, PVR, IIEF	TDF vs. SDS vs. TDF + SDS	Qmax, IPSS, PVR, and IIEF scores improved significantly more with the combination than with either drug alone.
Tawfik et al. [17]	258	IPSS, IPSS- QoL index, Qmax, Qave, IIEF	TMS + FNS vs. TDF + FNS	Both groups had significant IPSS changes. TMS + FNS had better Qave. Qmax was comparable in both groups at the 12th week. All IIEF domains were significantly lowered in the TMS + FNS group. TDF + FNS showed a significant increase in IIEF-erectile function scores.
Casabé et al. [18]	695	IPSS, IIEF-5	FNS +placebo vs TDF + FNS	TDF + FNS coadministration improves IPSS, and IIEF-5 in men who have comorbid erectile dysfunction.
Kosilov et al. [19]	285	IPSS, OABq, IIEF, MSHQ-EjD	DTS + SDS + TDL 0.5 + 2.5 + 2.5 vs. 0.5 + 5 + 5 vs. 0.5 + 10 + 20	The 0.5 + 10 + 20 Group had significant improvement in OABq after the fourth week of the study.
Gotoh et al. [20]	44	IPSS, QoL, NTUF, NTMVVOABSS, SHIM	TDF as add-on to DTS	IPSS, QoL, NTUF, NTMVV improved significantly at 4 weeks OABSS, SHIM improved at 12 weeks Qmax, PVR showed no improvement.
Yamanishi et al. [21]	24	OABSS, NTUF, NIH-CPSI, MC	TDF + MGB vs. TDF	Changes from baseline in OABSS, NTUF, NIH-CPSI, MC were significantly reduced in combination therapy.

(IPSS) International Prostate Symptom Score; (IPSS-QoL) IPSS Quality of Life; (Qmax) maximum flow rate; (Qave) average flow rate; (PVR); post-void residual volume; (IIEF-5) International Index of Erectile Function; (IIEF-EF) International Index of Erectile Function-Erectile Function Domain; (GAQ) Global Assessment Quality; (OABSS) Overactive bladder symptom score; (SHIM) sexual health inventory for men; (MSHQ-EjDQ) Male Sexual Health Questionnaire Ejaculatory Dysfunction; (OABq) Overactive Bladder Questionnaire; (NTUF) night-time urinary frequency; (NTMVV) night-time maximum voided volume; (MC) micturition chart; (TMS) tamsulosin; (TDF) tadalafil; (VRF) vardenafil; (SLD) Sildenafil; (SDS) Silodosin; (DTS) Dutasteride; (FNS) Finasteride; (MGB) mirabegron.
